# Experimental study of expression profile and specific role of human microRNAs in regulating atrophic bone nonunion

**DOI:** 10.1097/MD.0000000000021653

**Published:** 2020-09-04

**Authors:** Junqiang Wei, Hua Chen, Yangmu Fu, Boxun Zhang, Lihai Zhang, Sheng Tao, Feng Lin

**Affiliations:** a Department of Orthopedics, Hainan Hospital of Chinese People's Liberation Army General Hospital, Sanya; b Department of Orthopedics, Chinese People's Liberation Army General Hospital, Beijing, China.

**Keywords:** atrophic bone nonunion, microarray, miRNA, regulation, target gene

## Abstract

The expression profile and specific roles of microRNAs (miRNAs) in regulation of atrophic bone nonunion are not fully understood. Here, we present evidence that miRNAs are involved in regulation of several osteogenic genes and may contribute to the development of atrophic bone nonunion.

The miRNA expression profile of repairing tissues in atrophic bone nonunion patients (group A) and in callus tissues from patients with healed fractures (group B) were quantitatively measured. microRNA microarrays were used to identify differentially expressed miRNAs, and the bioinformatics methods were used to predict the potential target genes. Quantitative real-time polymerase chain reaction (qRT-PCR), western blot, and dual-luciferase reporter assay were performed in human bone marrow stromal cells (hBMSCs) to validate the microarray results.

Nine miRNAs in group A were up-regulated 1.5 times compared to group B, while the other 9 miRNAs in group A were down-regulated 1.5 times. Several target regions of these miRNAs were identified in the osteogenic genes, as well as in the other genes in their families or related regulatory factors. Four miRNAs (hsa-miR-149^∗^, hsa-miR-221, hsa-miR-628-3p, and hsa-miR-654-5p) could play important roles in regulating bone nonunion development. hBMSCs transfected with these miRNAs significantly decreased mRNA levels of alkaline phosphatase, liver/bone/kidney (*ALPL*), platelet derived growth factor subunit A (*PDGFA*), and bone morphogenetic protein 2 (*BMP2*). Lower protein expression levels were observed using western blotting, confirming that *ALPL*, *PDGFA*, and *BMP2* were directly targeted by hsa-miR-149^∗^, hsa-miR-221, and hsa-miR-654-5p, respectively.

In summary, hsa-miR-149^∗^, hsa-miR-221, and hsa-miR-654-5p may play important biological roles by repressing osteogenic target genes *ALPL*, *PDGFA*, and *BMP2*, and, therefore, contributing to progression of atrophic bone nonunion.

## 1 Introduction

miRNAs were first discovered and reported as small single-stranded non-coding RNAs of approximately 21 to 25 nucleotides in length. miRNAs function as regulators of gene expression either through translation repression by binding partially complementary 3’-untranslated regions (3’-UTR) of specific target mRNAs, or via induction of mRNA cleavage by pairing with fully complementary related mRNAs.^[1]^ The precursors of miRNAs are located in highly conserved genomic non-coding regions, the regions known to play key roles in development and differentiation.^[2]^ miRNAs are ubiquitously expressed and, at the same time, are tissue-specific. Numerous studies^[3,4]^ have shown that miRNAs are involved in regulation of various ubiquitous biological processes, such as developmental timing and patterning, bilateral asymmetry, differentiation, proliferation, morphogenesis, and apoptosis.^[5]^

More than 1000 miRNAs that have been identified in human species;^[6]^ however, little is known about the roles of miRNAs in fracture healing or bone nonunion. For example, the first study investigating miRNA expression during osteoblast differentiation was published only in 2008.^[7]^ Kim et al demonstrated that miR-196a regulates proliferation and osteogenic differentiation of human adipose tissue-derived mesenchymal stem cells, without affecting adipogenic differentiation.^[8]^ Another study^[9]^ profiled the expression of miRNAs during BMP2-induced osteogenesis in C2C12 mesenchymal cells, and showed that 22 out of 25 miRNAs were down-regulated in response to the BMP2 treatment. Mizuno et al demonstrated that miR-125b was involved in osteoblastic differentiation by regulating cell proliferation via the target genes, such as *ERBB2*.^[10]^ These studies focused primarily on osteogenic differentiation of stem cells; however, the biological roles of miRNAs in bone nonunion were not investigated.

Bone nonunion is a serious complication of bone fracture healing with the incidence rates between 5% and 10%, and these rates are even higher in patients with severe open fractures and soft tissue injuries.^[11]^ For example, a study of femoral and tibia shaft fractures demonstrated that around 25% of these fractures developed delayed union or nonunion.^[12]^ Although multiple therapeutic methods have been utilized,^[11,13]^ the specific molecular mechanisms underlying bone nonunion, especially the atrophic bone nonunion, are not understood, resulting in low clinical efficacy and high medical costs. Previous research demonstrated that the expression levels of bone morphogenetic proteins (BMPs) and BMP antagonists were significantly down-regulated in bone nonunion compared to the conventional healing fractures, suggesting that this low expression of osteogenic BMPs may be responsible for the nonunion of bone fracture.^[14]^ In addition to BMPs, growth differentiation factors and vascular endothelial growth factor, have been shown to promote bone healing;^[15]^ however, the roles of differentially expressed miRNAs and their specific regulatory molecular mechanisms in atrophic bone nonunion have not been fully investigated.^[16,17]^

In this study, we utilized bioinformatics methods to predict the potential target genes related to fracture healing and bone nonunion. The relationship between up-regulated miRNAs and their predicted target genes was validated in hBMSCs transfected with 4 types of synthetic double-stranded small RNAs to confirm the specific roles and molecular mechanisms of up-regulated miRNAs in the atrophic bone nonunion.

## 2 Methods

### 2.1 Tissue collection and processing

**Figure 1:**
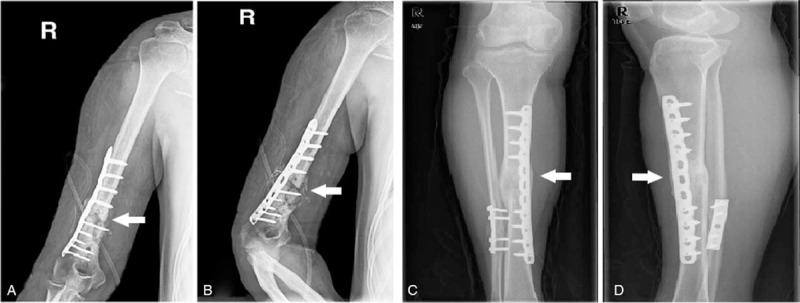
X-ray images of patients from 2 different groups. (A), (B) images of atrophic bone nonunion patient (anteroposterior view and lateral view); white arrows indicate atrophic bone nonunion location; (C), (D) images of fracture-healed patient (anteroposterior view and lateral view); white arrows indicate callus tissues.

Patients were enrolled by randomization. All tissue samples used in this study were surgically collected intraoperatively from the nonunion tissues of atrophic bone (nonunion patients, group A, n = 3) and from the callus tissues around the internal fixation plates (fracture-healed patients, group B, n = 3) (patient information is summarized in Table 1, sites of tissue collection are depicted in Fig. 1). The collected tissues were cut into small pieces, rinsed in physiological saline, immediately placed into the freezing tubes, and stored in liquid nitrogen under sterile conditions to avoid RNA degradation. Written informed consents were obtained from all patients. The study procedures were approved by the ethics committee of the Chinese People's Liberation Army General Hospital. The study adhered to the standards established by the Declaration of Helsinki.^[18]^

**Table 1:**
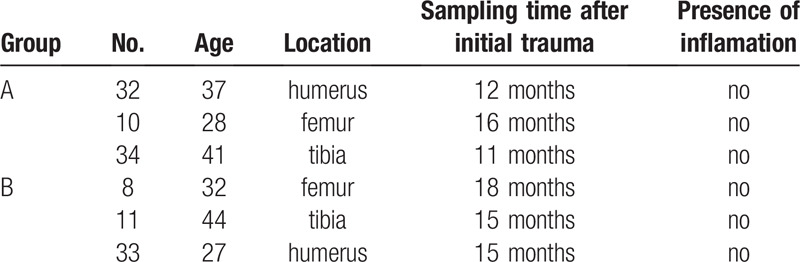
Patients data in 2 different groups.

### 2.2 Total RNA isolation and quality testing

Fifty to 100 mg of tissues were taken from each sample, frozen in liquid nitrogen, and crushed using the BioPulverizer^TM^. Next, the samples were homogenized using Mini-Bead-Beater-16 in 1 ml of the Trizol reagent (Invitrogen, U.S.) and the total RNA was extracted according to the manufacturers instructions. The absorption was measured at 260 nm, 280 nm, and 230 nm using the Nanodrop spectrophotometer (NanoDrop Technologies Inc., Wilmington, DE), and the total RNA concentration and purity were calculated. The quality of RNA was evaluated using the denaturing agarose gel electrophoresis.

### 2.3 miRNA microarray

miRCURY LNA miRNA Hy3/Hy5 Power labeling kit was used for labeling total RNA samples according to the manufacturers instructions. The labeled probes were hybridized to the miRCURY LNA miRNA Arrays (version 11.0) using heat shrinkable Phalanx^TM^ bags under standard conditions according to the manufacturers instructions. Microarray fluorescence intensity data were acquired using the Axon GenePix 4000B microarray scanner.

### 2.4 Intensity analysis

The raw intensity data were analyzed using the GenePix Pro Microarray Analysis Software (V6.0). To determine the intensity of green signal, background was subtracted and the median of 4 replicate spots for each probe on the same slide was calculated. Median normalization method was used to obtain “normalized data” as follows: “normalized data” = (foreground–background)/median, and the median is 50% quantile of miRNA intensity which is larger than 50 in all samples after background correction. The statistical significance of differentially expressed miRNAs was analyzed by *t* test.

### 2.5 Bioinformatics analysis of target genes

Predication of computational biology indicated that the regulations of miRNAs on target genes were extensive and complex. For example, the second-generation Oligo Osteogenesis microarray included 113 bone regeneration- and differentiation-related genes. Therefore, the aberrant expression of genes belonging to the same families as the osteogenic genes would also affect bone formation. To study possible relationships between the differentially expressed miRNAs and the osteogenesis genes, 2 databases (miRBase: http://microrna.sanger.ac.uk/targets/v5; miRanda: http://www.microrna.org) and several research articles were reviewed to predict the target genes.

### 2.6 hBMSC isolation, culture, and transfection

Human bone marrow blood was collected, hBMSCs were separated by centrifugation, and subcultured in DMEM (Life Technologies) containing 10% fetal bovine serum (HyClone, Logan, UT, USA) and 1/1000 double antibiotics at 37°C in 5% CO_2_ in a humidified incubator. The culture medium was replaced every 2 days. Cells between passages 1 and 4 at 90% confluency were passaged by trypsinization. Passage 4 cells were stained with Giemsa staining.^[19]^ Cells with and without staining were observed under a microscope and the images were captured using Nikon camera. Cell transfections were performed using Lipofectamine 2000 (Life Technologies) according to the manufacturers instructions. Briefly, cells were seeded in the 6-well plates, cultured to 70% to 80% confluence, and then washed twice with DMEM. Next, a transfection solution containing Lipofectamine 2000 (Life Technologies) and a miRNA mimic (miR-149^∗^, miR-221, miR-628-3p, miR-654-5p) or negative controls (GenePharma, Shanghai, China) were added to the wells and incubated for 48 hours.

### 2.7 qRT-PCR for miRNA and mRNA validation

Total RNA was extracted from the tissues and cultured cells using the TRIzol reagent (Life Technologies) according to the manufacturer's instructions. A total of 0.5 μg of RNA, MMLV reverse transcriptase, RNase inhibitor (for miRNAs, Epicentre, USA), and ImProm-II reverse transcriptase (for mRNAs, Promega, USA) were used for reverse transcription. qPCR was performed using a SYBERPremix TaqTM II kit (Takara) and 2 μl of the cDNA template in a final reaction mixture (95°C for 10 minutes; 95°C for 10 seconds, 60°C for 40 seconds, 72°C for 30 seconds, 40 cycles) in an Mx3005P real-time PCR system (Agilent Stratagene, USA). The average threshold cycle (Ct) for each gene was determined from triplicate reactions; the relative expression levels of miRNAs (miRNAs and corresponding sequences are summarized in Table 2) and mRNAs were normalized to the internal controls, glyceraldehyde-3-phosphate dehydrogenase (GAPDH, for qRT-PCR of mRNAs) or U6 (for qRT-PCR of miRNAs), using the 2^–ΔΔCt^ method.^[20]^ Predicted target genes and their mRNA primer sequences are summarized in Table 3. The primers for GAPDH were 5’-TCAGTGGTGGACCTGACCTG-3’ (sense) and 5’-TGCTGTAGCCAAATTCGTTG-3’ (antisense), and for U6 were 5’-GCTTCGGCAGCACATATACTAAAAT-3’ (sense) and 5’-CGCTTCACGAATTTGCGTGTCAT-3’ (antisense).

**Table 2:**
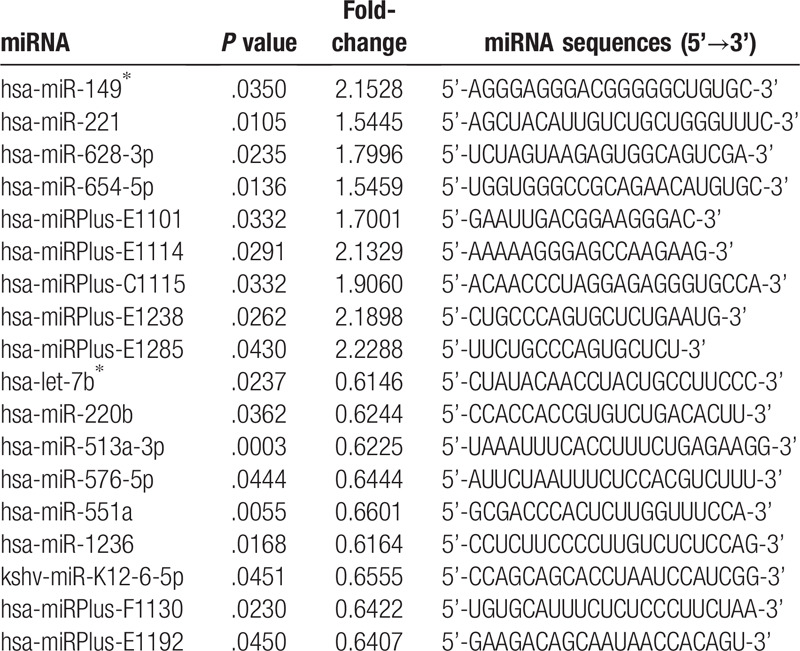
Differently expressed miRNAs in atrophic bone nonunion tissues.

**Table 3:**
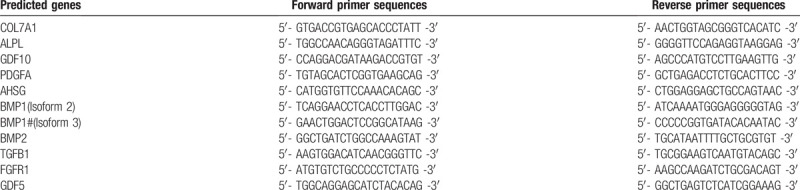
Predicted target genes to be tested and their mRNA primer sequences.

### 2.8 Western blot

The experimental procedures were performed as per a previously described method.^[21]^ Briefly, the cells were harvested and lysed with the RIPA lysis buffer. Separated proteins were transferred to 0.4-μm polyvinylidene difluoride (PVDF) membranes. The blots were blocked with 5% skim milk for 3 hours, and then incubated overnight at 4°C with one of the primary antibodies (ALPL (11187-1-AP, 300 μg/ml, Proteintech, China), PDGFA (E13750, 200 μg/ml, Spring Bioscience, USA), BMP2 (18933-1-AP, 250 μg/ml, Proteintech, China), or GAPDH (H00002597-M01) (1:1,000; Abnova, Taipei, China) diluted in blocking solution. The blots were washed 3 times with Tris-buffered saline with Tween-20 (TBST) and then incubated with horseradish peroxidase-conjugated secondary antibodies (ZB-2305, 80 μg/200 μl, ZSGB-BIO, Beijing, China) for 1 hour at room temperature. Next, the blots were washed twice with TBST, once with Tris-buffered saline (TBS), and then incubated in enhanced chemiluminescence (ECL) reagent for 5 minutes. Protein bands were visualized using the Western Blotting Systems ECL kit (Amersham, Piscataway, NJ, USA). Image J software was used to qualify the scanned results.

### 2.9 Luciferase reporter assay

The experimental procedures were performed as per a previously described method.^[21]^ Briefly, the 3’UTR luciferase reporter constructs of ALPL, PDGFA, and BMP2 were made by cloning the 3’UTR region of the mRNAs into the pGL-3M-promoter vector (Promega, Madison, WI, USA). hBMSCs were co-transfected with the following luciferase reporter plasmids: pGL-3M-ALPL, pGL-3M-ALPLm1 (mutated first target site), pGL-3M-ALPLm2 (mutated second target site), pGL-3M-ALPLm (mutated both first and second target sites), pGL-3M-PDGFA, pGL-3M-PDGFAm, pGL-3M-BMP2, and pGL-3M-BMP2m. pRL-CMV was used as the second plasmid for the luciferase report assay. After 24 hour, the luciferase activities were measured using the dual-luciferase reporter assay system.

## 3 Results

### 3.1 miRNA expression profile

**Figure 2:**
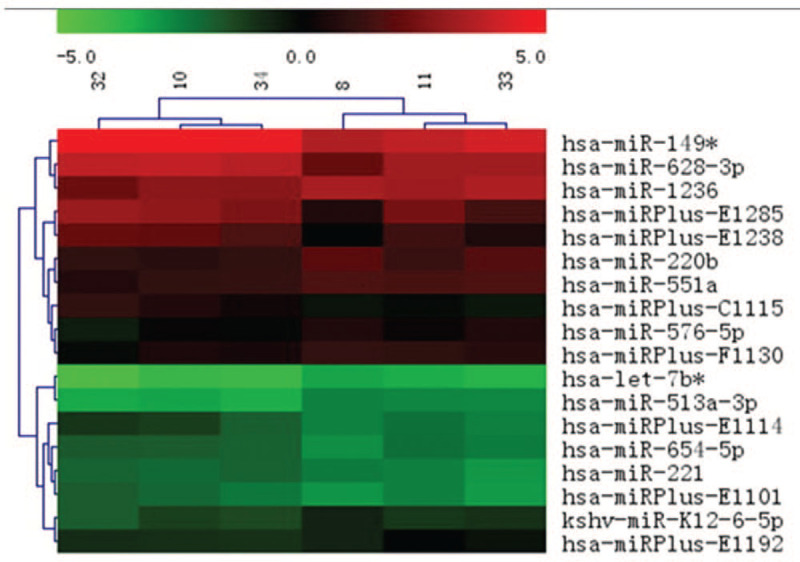
Hierarchical clustering map of differentially expressed miRNAs. Rows represent miRNAs and columns represent specimens. Three specimens on the left are from atrophic bone nonunion patients (group A) and 3 specimens on the right are from the fracture-healed patients (group B). The miRNA clustering tree is shown on the left, and the sample clustering tree is depicted at the top. The color scale depicts relative expression levels of miRNAs: red represents high expression levels, and green represents low expression levels.

Microarray results demonstrated that 113 miRNAs were differentially expressed between the atrophic bone nonunion (group A) and fracture-healed (group B) samples, therefore, separating these samples into biologically relevant groups. Nine miRNAs in group A were up-regulated more than 1.5-fold (*P* < .05) compared to group B, including hsa-miR-149^∗^, hsa-miR-221, hsa-miR-628-3p, hsa-miR-654-5p, and 5 plus hsa-miRNAs (hsa-miRPlus-E1114, hsa-miRPlus-E1285, hsa-miRPlus-E1238, hsa-miRPlus-E1101, hsa-miRPlus-C1115; these plus hsa-miRNAs have been predicted, but not annotated in miRBase, and, therefore, little information is available about these miRNAs). Another 9 miRNAs in group A were down-regulated less than 0.67-fold (*P* < .05), including hsa-let-7b^∗^, hsa-miR-220b, hsa-miR-513a-3p, hsa-miR-551a, hsa-miR-576-5p, hsa-miR-1236, kshv-miR-K12-6 -5p, as well as 2 plus hsa-miRNAs (hsa-miRPlus-F1130, hsa-miRPlus-E1192), as illustrated in Figure 2 and summarized in Table 2.

### 3.2 hBMSC isolation, culture, and transfection

**Figure 3:**
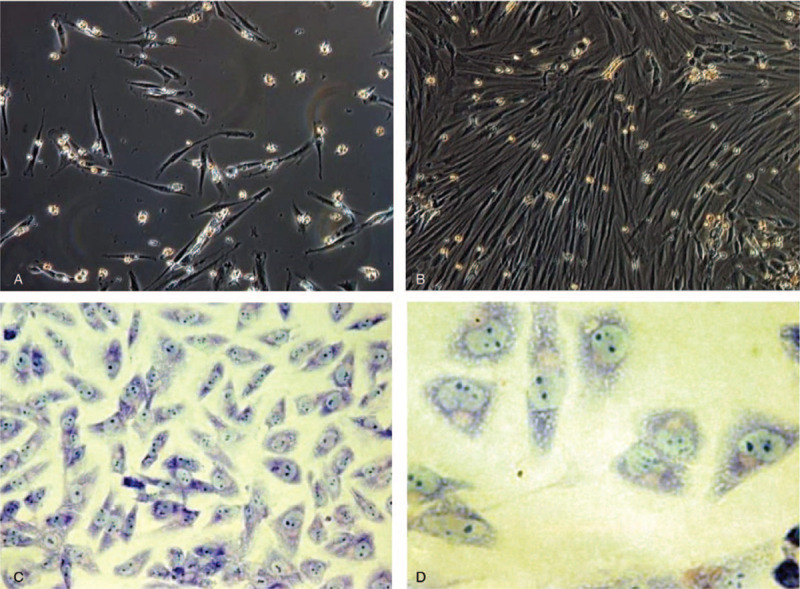
(A) hBMSCs morphology after replacement of culture medium (100×); (B) pictures after cell purification (100×); (C) (Giemsa staining, 200X); (D) (Giemsa staining, 400×). Giemsa staining revealed that hBMSCs assumed a shuttle shape with large nucleus and abundant cytoplasm. BMSCs actively grew and proliferated.

After the first replacement of culture medium, a large number of suspended hBMSCs were seen in scattered spindle or short-spindle shape. After 7 days, the adherent cells were distributed in clusters with fusiform shape, whereas mixed cells continued to exist. In the early stage, most cells grew into a fusiform, closely and evenly distributed. The passage cells were large and transparent in round shape. After 12 hours, the cells basically adhered to the wall and assumed a shuttle shape. At 2 to 4 days, the cells were rapidly proliferated in uniform and long fusiform. At 5 to 6 days, the cells became confluent and closely distributed in one direction. No significant difference was observed in terms of the time to adhere to the flask wall, proliferation and cell morphology among the 1st and 4th generations of cells. At the beginning of each generation, there are scattered fusiform cells adhered on the flask wall, and the suspended cells gradually adhere to the wall. At the end of each generation, after culture medium were changed several times, the cells became long fusiform shapes, closely arranged, evenly distributed and proliferated actively (Fig. 3).^[21,22]^

### 3.3 qRT-PCR

**Figure 4:**
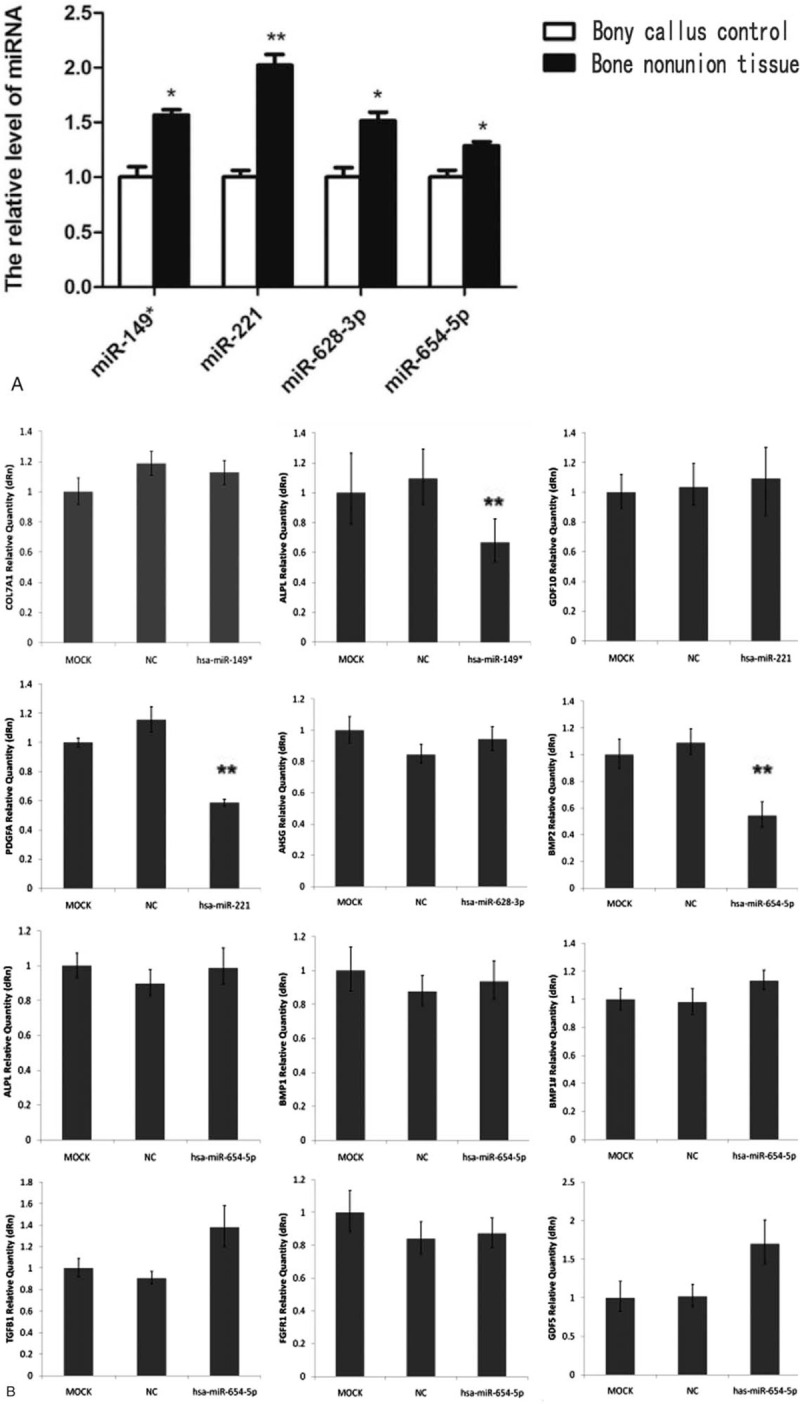
(A) qRT-PCR validation of 4 up-regulated miRNAs identified during the miRNA microarray screen. White bars represent the bony callus control group and black bars represent the bone nonunion tissue group. ΔCt values obtained from qPCR were subjected to a paired *t* test (ΔCt = Ct miR - Ct U6). The expression levels of miR–149^∗^, miR–221, miR–628-3p, and miR–654-5p at the site of bone nonunion are significantly higher compared to the bony callus tissues. ^∗^*P* < .05 and ^∗∗^*P* < .01 compared to the controls. (B) qRT-PCR evaluation of predicted target genes of 4 up-regulated miRNAs. hBMSCs were transfected with synthetic double-stranded small RNAs mimicking the sequences of 4 up-regulated miRNAs for 48 hours, and the mRNA expression levels of 11 predicted target genes were determined by qRT-PCR. The expression levels of *ALPL* (predicted target gene of hsa-miR-149^∗^), *PDGFA* (predicted target gene of hsa-miR-221), and *BMP2* (predicted target gene of hsa-miR-654-5p) are significantly decreased; significant differences are not observed for the other genes, including *AHSG*, the predicted target gene of hsa-miR-628-3p.

The four miRNAs up-regulated in patients with atrophic nonunion were validated by qPCR. Relative quantification was performed using the 2^–ΔΔCt^ method, and presented as a ratio to the bony callus samples (Fig. 4A). ΔCt values obtained from qPCR (ΔCt = Ct miR - Ct U6) were subjected to a paired *t*-test. The expression levels of miR-149^∗^, miR-221, miR-628-3p, and miR-654-5p from the bone nonunion samples were significantly higher compared to the miRNA levels from the bony callus tissues.

The cultured hBMSCs displayed stem cell-like characteristics, adhered to the flask wall, proliferated and integrated easily, and differentiated into the osteogenic cells. Therefore, hBMSCs were selected for the validation experiments. Synthetic double-stranded small RNAs mimicking the sequences of 4 up-regulated miRNAs were transfected into hBMSCs. The qRT-PCR data indicated that the corresponding miRNAs expression levels were significantly increased, while the mRNA expression levels of *ALPL* (predicted target gene of hsa-miR-149^∗^), *PDGFA* (predicted target gene of hsa-miR-221), and *BMP2* (predicted target gene of hsa-miR-654-5p) were significantly decreased (Fig. 4B). There was no effect on the expression levels of other predicted target genes. For example, the only predicted target gene for hsa-miR-628-3p (out of 113 bone regeneration and differentiation-related genes) was *AHSG*; however, the overexpression of hsa-miR-628-3p in our system did not have an effect on *AHSG* gene expression levels. Therefore, the role of this particular miRNA in bone nonunion still remains to be elucidated.

### 3.4 Western blot

**Figure 5:**
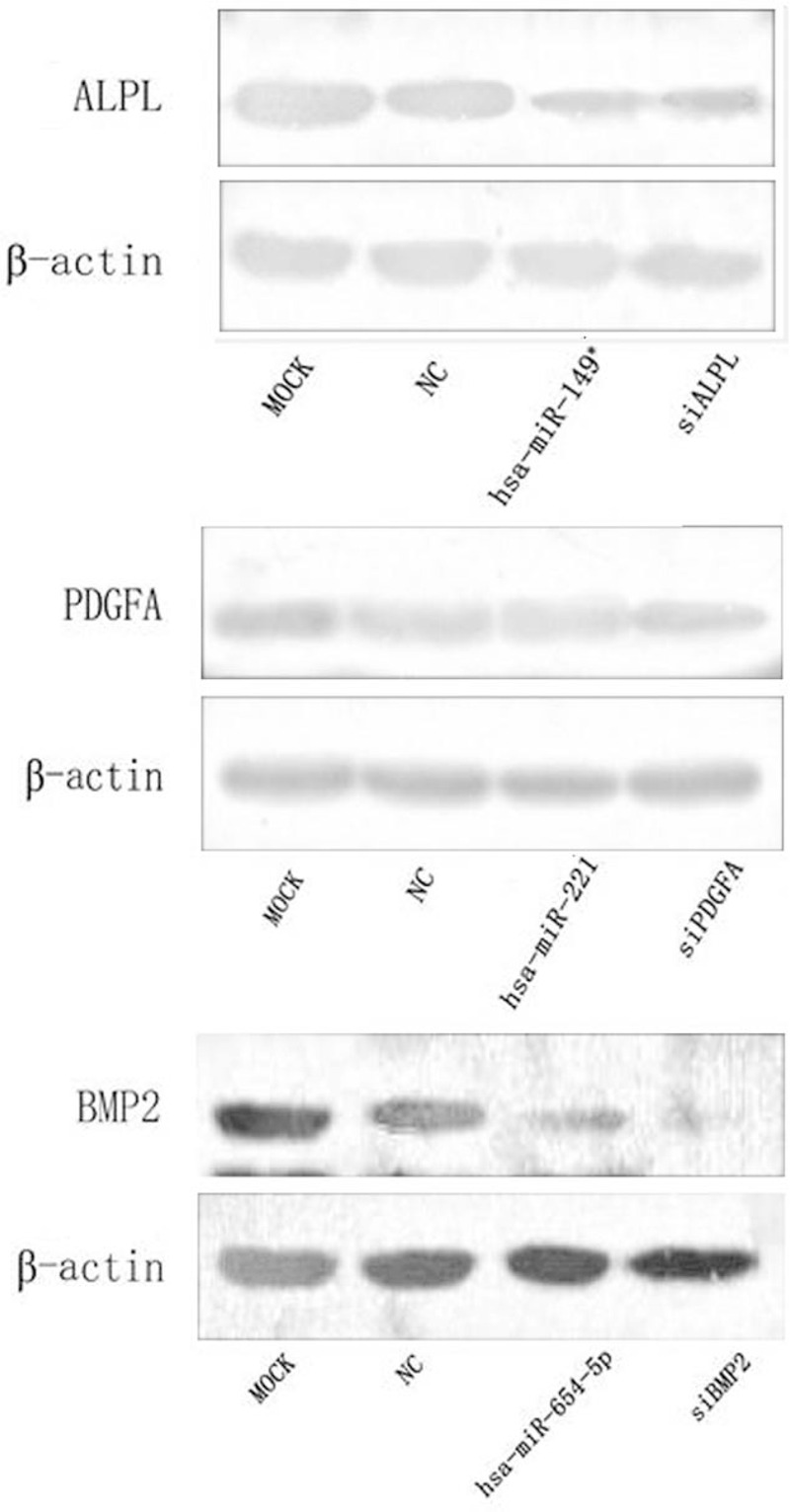
Western blot of ALPL, PDGFA, and BMP2 proteins. Representative images are shown. Protein levels of ALPL, PDGFA, and BMP2 proteins appear to be suppressed by overexpression of corresponding miRNAs.

The protein levels of ALPL, PDGFA, and BMP2 were significantly decreased after transient transfection of hBMSCs with hsa-miR-149^∗^, hsa-miR-221, hsa-miR-654-5p; double stranded RNA and small interfering RNA (siRNA) were used as a negative control (NC) and a positive control, respectively (Fig. 5). These results suggested that overexpression of corresponding miRNAs down-regulated the protein levels of ALPL, PDGFA, and BMP2.

### 3.5 Luciferase reporter assay

**Figure 6:**
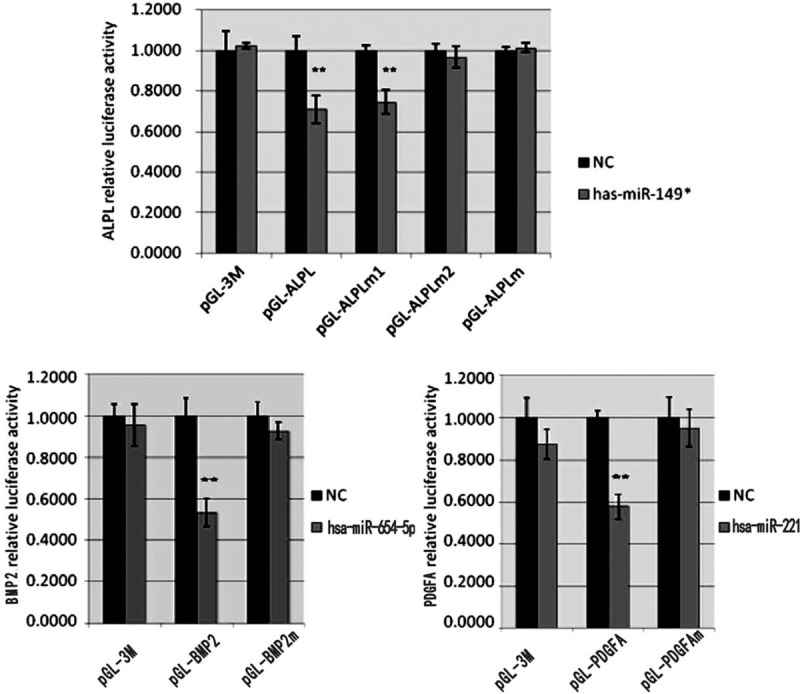
Luciferase reporter assay. The expression levels of *ALPL*, *PDGFA*, and *BMP2* mRNA show significantly inhibition by overexpression of corresponding miRNAs.

We already demonstrated that the expression levels of *ALPL*, *PDGFA*, and *BMP2* mRNA were significantly decreased after overexpression of corresponding miRNAs (Fig. 4). To confirm the specificity of the miRNAs, next, we co-transfected the cells with the miRNAs, as well as with plasmids containing a mutated predicted target site for each miRNA. As expected, the luciferase activity in cells transfected with the mutated *PDGFA* and *BMP2* was similar to the control levels, confirming that hsa-miR-221 and hsa-miR-654-5p directly target *PDGFA* and *BMP2* (Fig. 6). For *ALPL*, only the second target site mutation had rescued mRNA expression, while the first target site mutation had similar expression levels to the wild-type, suggesting that hsa-miR-149 affected gene expression directly via the second predicted site. In summary, luciferase reporter assay confirmed that hsa-miR-149^∗^, hsa-miR-221, and hsa-miR-654-5p can directly inhibit gene expression of corresponding target genes and, therefore, could negatively regulate osteoblast differentiation and function.

## 4 Discussion

In this investigation, we compared the miRNA expression profiles of tissues from the atrophic bone nonunion patients to the callus tissues of patients with healed fractures using the Exiqon miRCURY LNA miRNA microarrays. Our results demonstrated that the repairing tissues of atrophic bone nonunion patients had a different miRNA expression profile compared to the callus tissues of patients with healed fractures. These differentially expressed miRNA profiles were consistent for each specimen belonging to the same group and the overall concordance between the samples of group A and group B was high. Therefore, with the optimized RNA isolation, data normalization, and microarray methodologies, miRNA expression profiling of bone nonunion repairing samples is a robust and accurate process appropriate for assessing the miRNA expression changes. qRT-PCR verification in both tissues and hBMSC levels showed a different miRNA expression profile between 2 groups.

This study brings a new perspective to the investigation of molecular mechanisms of fracture healing and the atrophic bone nonunion and the roles that miRNAs play in this process.^[23]^ It was previously demonstrated that the down-regulated expression of osteogenic genes was responsible for the nonunion, and not the up-regulated expression of antagonists.^[11]^ To elucidate the mechanism of down-regulated target genes in atrophic bone nonunion, we decided to focus on up-regulated levels of miRNAs, since miRNAs are responsible for the translational repression and/or mRNA cleavage by the miRNA-induced silencing complexes.^[13]^ Our results showed that the up-regulated miRNAs, such as hsa-miR-149^∗^, hsa-miR-221, hsa-miR-628-3p, and hsa-miR-654-5p, could play pivotal roles in the development and regulation of atrophic bone nonunion by repressing several osteogenic target genes. Our findings were validated by the bioinformatic analysis, qRT-PCR for miRNA and mRNA, western blot, as well as the luciferase reporter assay.

The potential functional target genes of differentially expressed miRNAs identified in our screen include osteogenic genes and the genes of related regulatory factors involved in initiation of fracture repair. BMPs are multi-functional growth and differentiation factors that belong to the transforming growth factor-beta (TGF-β) superfamily.^[24]^ TGF-β has been proposed to have a role in bone remodeling by affecting the differentiation and activity of bone-forming osteoblasts and bone-resorbing osteoclasts. BMP-2 is the major bone morphogenetic protein used in preclinical and clinical studies to enhance osteoblasts differentiation and function, and, therefore, may be applied to treat bone defects, nonunion fractures, spinal fusion, osteoporosis, and during a root canal surgery.^[25]^ ALPL and bone gamma-carboxyglutamate protein are markers of mature osteoblasts, and the expression of these proteins correlates with bone formation and calcification.^[11–15]^ PDGFs are mitogens affecting both osteoblast and osteoclast lineages, and treatment with PDGF could selectively stimulate proliferation and function of fibroblasts in the early stages of fracture healing to improve bone formation.^[26]^

This study had a limited sample size (n = 6), therefore, the results and conclusions need to be further validated. Nevertheless, we predicted and validated interactions of miRNAs with their potential target genes using the data from miRBase and miRanda databases, and our future in vitro and in vivo experiments will focus on confirming and expanding these findings.

## 5 Conclusion

In summary, we identified several miRNAs that could play key regulatory roles during tissue healing in atrophic bone nonunion. We convincingly demonstrated that 3 significantly up-regulated miRNAs suppressed the osteogenic target genes, suggesting a possible mechanism responsible for the development of atrophic bone nonunion.

### 5.1 Uncited reference

^[8]^.

## Author contributions

**Conceptualization:** Hua Chen.

**Data curation:** Boxun Zhang.

**Funding acquisition:** Hua Chen, Junqiang Wei.

**Methodology and investigation:** Sheng Tao, Feng Lin.

**Writing – original draft:** Junqiang Wei.

**Writing – review & editing:** Yangmu Fu.
